# Service Engagement: Psychopathology, Recovery Style and Treatments

**DOI:** 10.1155/2014/249852

**Published:** 2014-02-12

**Authors:** Simone Vender, Nicola Poloni, Francesca Aletti, Cristiano Bonalumi, Camilla Callegari

**Affiliations:** Department of Clinical and Experimental Medicine, Psychiatry, Faculty of Medicine and Surgery, University of Insubria, Via Rossi No. 9, 21100 Varese, Italy

## Abstract

The aim of the present study is to evaluate how recovery style, a set of strategies used by patients to interact with services and therapists, and the severity of psychotic symptoms affect the quality/continuity of taking charge of each patient. 156 psychotic patients at different stages of illness were enrolled. Sociodemographic and clinical data were collected and integration/sealing-Over Scale, Recovery Style Questionnaire and Positive and Negative Syndrome Scale were administered. Patients were distinguished into four groups according to the type of treatment received: clinical package, hospital package, day-care package, and residential package. A positive correlation between the cost of psychiatric performance and psychopathological severity (measured with PANSS scores) was identified. No association emerged between ISOS/RSQ total scores and costs. The sanitary expenditure appears to be linked to positive psychotic symptoms while lower performances are given for the treatment of patients with predominant negative symptoms. Recovery style itself has not a direct influence on the quantity/quality of psychiatric services.

## 1. Introduction

The turn of the century has seen a number of changes in perceptions of mental disorder, treatment approaches and goals, and mental care systems [1]. It is now crucial to understand whether a patient, in particular if affected by psychotic disorder, will adhere to treatment sufficiently to provide therapeutic benefits. Schizophrenia, delusional disorder, schizoaffective disorder, and mood disorders with psychotic symptoms are diseases characterized by a loss of contact with reality, a fracture in experiential continuity, an impairment of ego functions as well in the personal and social functioning. The concept of service engagement emerges in the psychiatric literature in the early 1990s: until the 1980s most work on patients' engagement with medication regimes was described as compliance. This expression has fallen out of favour in clinical practice because of the increasing concern that the term carries an assumption that patients are passive recipients of the doctors and that the clinician is always in a position of authority. In recent years there has been a shift away from this paternalistic model of doctor-patient interaction with the consequent preference for the use of the term adherence, advocating that the focus should be on concordance. It is also important to consider that the patient and the professional may legitimately hold differing ideas about what would be the appropriate treatment intervention and that the management of any symptoms or disorder will require negotiation [2].

Patients using modern health systems are more active in their own health care so that treatment regimes are modified or distorted by the consumer rather than being completely accepted or totally abandoned. Thus, the notion of adherence (patient acceptance of an engagement in healthy behaviours) incorporates the idea that this behaviour is in a dynamic state and may change over time [3]. Moreover, the therapeutic alliance is not a dual relationship between an operator and his patient, because it often involves health professionals and links to specific environments, experiences, and memories.

The methods used to measure nonadherence have been wide ranging and the actual approach employed is partly dictated by the setting in which measurements takes place, the financial and personal resources available to undertake the assessment and the acceptable response burden placed on the patient [4]. Subjective methods include examining case-note recordings, direct patient interviews, and obtaining collateral reports from clinicians or significant others, [5, 6]. Objective measurements of adherence include monitoring rates of dispensing of repeat prescriptions, counting the number of tablets left in the pill bottles, monitoring of serum drug levels, and analysis of urine for drugs or their metabolites, [7]. The current state of the art usually involves a combination of approaches but all these assessments give an estimate of the patient's behaviour rather than a definitive measurement of adherence [8].

Despite the objective difficulty to quantify the service engagement, psychometric evaluation methods are however born, such as Working Alliance Inventory [9], Active Engagement Scale [10], Service Engagement Scale [11], and Singh O'Brien Level of Engagement Scale [12]. Startup et al. [13] tried to extend the definition of service engagement with focus on the attitudes of the patient defined in terms of availability for appointments, collaboration with mental health professionals, help-seeking, and treatment adherence.

The Italian validation of the Integration/Sealing-Over Scale (ISOS) and the Recovery Style Questionnaire (RSQ) [14] allowed to evaluate the impact of recovery style on the prognosis and on the involvement with psychiatric services. McGlashan et al. identified two main recovery styles: “sealing-over,” in which the subject minimizes and tends to remove the recent psychotic episode, and “integration,” in which there is a continuity between psychotic and pre/post-psychotic experiences [15–19].

Using the RSQ, Tait et al. [20] studied how insight, psychotic symptoms and recovery style may predict patient's involvement with psychiatric services, recognizing that the tendency to sealing-over is associated with a service engagement considerably lower than integration. The same authors recognized as the sealers have attachment difficulties to care-givers [21].

Modestin et al. [22], examining the relationship between recovery style and positive and negative symptoms, found a negative correlation between the intensity of negative symptoms and integration. Therefore as the negative symptoms become more evident, the tendency to sealing-over increases, so that it can be assumed that the engagement is different.

The concept of service engagement is very broad and there is no gold standard universally accepted by the scientific community but rather attempts of several research groups to give a quantitative value to this variable.

The perspective of the service engagement tends to evaluate patients before treatment and then build a path to verify the accuracy of these assessments, perhaps foreshadowing a financial commitment. Do the recovery style and psychopathological severity predict a different engagement? Furthermore, has the recovery style itself an influence on the quantity/quality of psychiatric services?

Our study aimed at verifying the engagement by means set out in detail. The purpose of this research was to assess the relationship between recovery style, psychopathological symptoms, and quantity/quality of psychiatric annual performance (considered from an economic point of view) to assess differences in the service engagement of the patients.

## 2. Materials and Methods

### 2.1. Recruitment of Patients

This study tested a group of 156 patients treated in the Community Mental Health Center (CMHC) of Varese. It is located in an urban and high-industrialized part of the Lombardia Region of Italy and has a catchment area of about 180,000 inhabitants. Approximately 2400 outpatients are in charge (have at least one medical visit per year) of our CMHC.

We used a consecutive sampling over a period of 18 months (September 2011–February 2013) and we included patients with diagnosis of psychotic disorders (schizophrenia, delusional disorder, and schizoaffective disorder) and mood disorders with psychotic symptoms according to the International Classification of Diseases (ICD-10) criteria [23]. We excluded patients with a recent (≤6 months) acute psychotic episode of acute psychosis or at psychotic onset, mental retardation, dementia, and organic and neurological disorders.

The rating scales were administered in our outpatient services during follow-up appointments; 167 potential participants were recruited and 11 subjects did not agree to participate.

Psychotic patients are followed by CMHCs for very long periods while subjects with neurotic or personality disorders tend to drop out [24]. We would like to specify that our psychiatric services adopt a standard treatment for psychosis, although the care pathway is customized and includes a consultant psychiatrist and a case manager in more severe clinical cases.

Taking into account the type of treatment received we distinguished four groups of patients:

1. clinical package patients, treated only in the community by psychiatrists, psychologists, or other mental health professionals (such as nurses, social workers, and occupational therapists);
2. hospital package patients who, without any admissions to residential facilities, had at least one admission to the Psychiatric Inpatient Unit located in the General Hospital;
3. day-care package patients that receive day-care treatment in dedicated day centres (with or without any admission to hospital);
4. residential package patients who had at least an admission to a therapeutic community, independently of contacts with other facilities.

### 2.2. Assessment

The following rating scales were administered. 

*Integration/Sealing-Over-Scale (ISOS) [14–16]*. It is a 13-item scale derived from previous qualitative research to identify a rating of recovery style using a semistructured clinical interview. Integration and sealing-over are located at the extremes of a 6-point Likert scale, each point representing a different style. 

*Recovery Style Questionnaire (RSQ) [14, 18]*. It is a 39-item self-report measure, designed to reflect categories consistent with those developed by McGlashan et al. (1977). Four recovery styles can be classified: integration, mixed picture in which integration predominates, mixed picture in which sealing-over predominates, and sealing-over. Higher scores represent sealing-over. RSQ tends to overestimate the integration comparing to ISOS [14]. 

*Positive and Negative Syndrome Scale (PANSS) [25–27]*. It is based on findings that schizophrenia comprises at least two distinct syndromes: the positive one characterized by productive symptoms, and the negative one consisting of deficit features. The patient is rated from 1 to 7 on 30 different symptoms based on a 45-minute clinical interview as well as reports of family members or primary care hospital workers. The PANSS includes a Positive Scale (PANSS-P), a Negative Scale (PANSS-N), and a General Psychopathology Scale (PANSS-G).

### 2.3. Evaluation of Psychiatric Costs

The software PSYCHE of Lombardy region is an electronic register of services provided to patients in charge of the CMHC and it also allows the evaluation of economic benefits on the basis of specific rates established according to quality and location of intervention (i.e., inpatient, outpatient, or community treatment).

In Italy the economic values of hospital services are based on the diagnosis-related group (D.R.G.); instead specific rates for outpatient or community services are set according to performance. For example costs are directly proportional to the number of mental health professionals which are involved in the care pathway.

### 2.4. Procedures and Statistical Analysis

ISOS and PANSS were administered by a qualified psychiatrists and RSQ was completed with supervision.

The scales were administered in a single session in some cases at the end of a clinical interview while at other times during appointments dedicated only to psychometric evaluation. We then proceeded to the collection of sociodemographic data, diagnosis, and information about psychopathological features potentially related to recovery style (such as onset, first treatment age, disease duration, number of hospitalizations, and symptoms of the last acute psychotic episode). A database including sociodemographic variables and rating scales' scores was subsequently developed.

For descriptive analysis we calculated means, standard deviations, and percentage values. For comparisons between means we used *t*-test and analysis of variance (ANOVA); for the analysis of relationships between variables we operated the Pearson bivariate correlation.

All calculations were performed using SPSS 13 (SPSS Inc. Chicago, USA).

## 3. Results

The patients, 37.9% male and 62.1% female, had a mean age of 41,02 sd ± 10,64, 56.6% were unemployed, and 67.9% were unmarried; for what concerns the educational level 83% of the sample had secondary or high degree.

The subjects were grouped into two categories with regard to the diagnosis: 64.4% were suffering from Schizophrenia or other Psychotic disorders and 35.6% were affected by Mood disorders.

The diagnoses in descending order resulted to be: paranoid schizophrenia (35.8%), schizoaffective disorder (17%), bipolar affective disorder (9.4%), delusional disorder (5.7%), and psychosis NOS (5.7%).

With regard to disease onset it turned out to be insidious for 71.7% patients, disruptive for 15.1%, and “other” for 13.2% patients.

As regards the duration of the disease 24.7% of subjects had been treated from 1 to 5 years, 24.3% from 6 to 11 years, 22.9% from 12 to 17 years, and 28.0% over 18 years.

Women were more likely to adopt integration as recovery style and were more inclined to use MCHC.

Over 45-year patients tend to integrate the experience of illness within their life.

Contrary to expected there was no correlation between recovery style, marital status, and education level.

Patients with a diagnosis of Schizophrenia or related disorders adopt more frequently the sealing-over compared to those with affective disorder, which tend to integration; this last finding confirms previous existing data [17].

### 3.1. Recovery Style, Psychopathology, and Psychiatric Services

Different frequencies of recovery style patterns resulted from the evaluation of ISOS and RSQ scores. This finding was expected considering previous studies on this topic.

The average annual cost per patient resulted to be 5943,32 sd ± 6146,49 Euros. This result turned out to be similar to that reported in the study of Donisi et al. [28] in which the annual average cost for psychotic patients was 5388,87 sd ± 7827,96 Euros. After totting up all the rates for services provided annually to the patients, we calculated the average annual rates for the two types of recovery style (Table 1).

**Figure 1:**
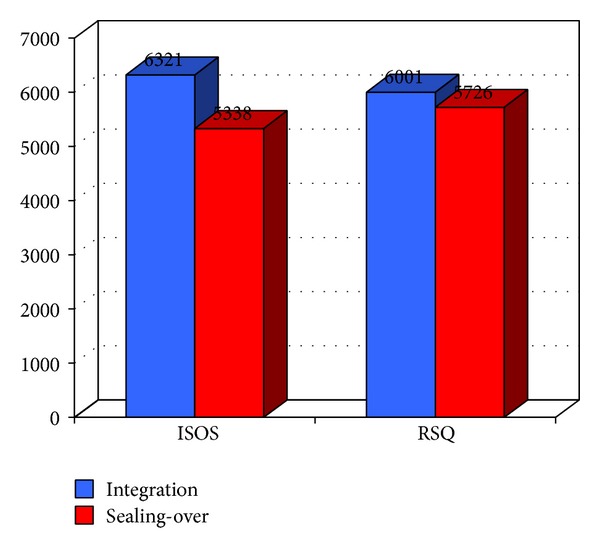
Comparison of average incurred costs for patients with different recovery style. The blue bar represents integrator subjects while the red bar represents sealing-over subjects. Integrator patients are provided with superior annual performance compared to sealing-over patients. Costs are expressed in Euros.

Table 1 and Figure 1 show how integrators are provided with superior annual performance compared to sealing-over patients. The standard deviation of the annual cost of services in the two groups is very high and for this reason *t*-test significance is not reached.

No differences were found in PANSS values between integrators and sealers.

Using bivariate Pearson correlation to assess relationship between annual costs per patient and ISOS/RSQ scores there was no statistically significant correlation. A positive correlation was instead identified between cost and PANSS-G (*P* < 0.001) and PANSS-P (*P* < 0.005) but not with PANSS-N (Table 2).

### 3.2. Service Engagement

As previously described, the sample was divided into four ranks of engagement. The ANOVA test was used to highlight differences in the four ranks between averages of the scales used (Table 3).

For what concerns PANSS mean scores, we highlighted a significant difference between ranks in PANSS-G (*P* < 0.05) and in PANSS-N (*P* < 0.05); the values of PANSS-G tend to grow consensually with engagement while the values of PANSS-N reach the highest average in group number 3 (day-care package patients).

Furthermore there was no difference in the means of recovery style scales (ISOS and RSQ).

**Figure 2:**
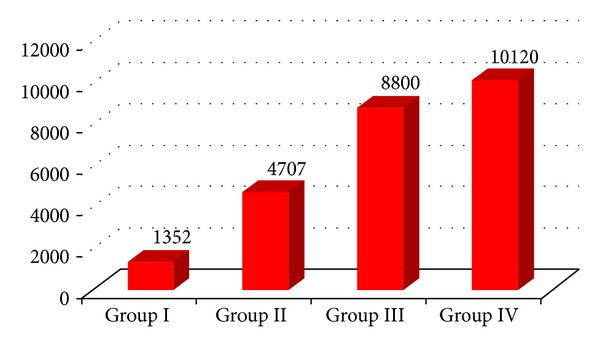
Average cost per patient in the four engagement groups. Costs are expressed in Euros.

With regard to the expenditure, a significant difference in mean costs (*P* = 0.001) was found, with a gradual increase in the patients with less engagement (only outpatient treatment) to those with greater commitment in services (residential rehabilitation treatment), as shown in Figure 2.

Applying Pearson bivariate correlation (Table 4), we found out an expected positive correlation between service engagement and annual costs (*P* < 0.001).

## 4. Discussion

In the selected sample the economic burden is primarily determined by positive (PANSS-P) and general (PANSS-G) psychotic symptoms. This finding is quite predictable as patients with positive symptoms need continuative care in the medium/long term. Severity of negative symptoms does not influence an increase in costs: patients with preeminent negative symptomatology indeed tend not to engage with services due to the social withdrawal typical of such a clinical condition. Numerous data emphasize that the schizophrenic functioning is adversely affected by the presence of negative symptoms [29, 30]. This finding appears to suggest that therapeutic environment is more effective in reducing negative symptoms than active engagement.

Moreover it was enlightened how the economic burden increases progressively depending on the service used by the patient, rising from the lowest value for outpatient users to the higher value for community users. This result seems consistent with the gradually increasing complexity of the interventions that are required for hospital, semiresidential, and residential treatment.

It was then considered the possibility that the distribution of patients between different services could depend either on the severity of psychopathology or on the recovery style. We can affirm that, considering our data, the latter parameter does not affect the treatment site. The only significant factor is the psychopathological condition assessed with the already mentioned rating scales.

Regarding the relationship between recovery style and cost, we found out that the average value of the expenses is moderately higher for integrators, although not statistically significant. This finding may reveal the importance in defining the recovery style adopted by the patient, as the positive or negative psychotic symptoms are likely independent from recovery style. Therefore, ISOS and RSQ, in addition to the PANSS measure, could help in predicting more precisely the economic burden.

Future studies should investigate this topic through the recruitment of a different and more numerous sample, in order to explore more deeply the mutual relationship between recovery styles, service engagement, and economic costs.

A last consideration arises in relation to the economic reporting system currently in use in Italy, in which the cost is determined by the individual services provided to the patient.

The economic burden for a single patient may be different if we could adopt a treatment plan based on individualized needs, previously defined by the diagnostic, clinical, and prognostic profile monitored with specific rating scales.

## 5. Conclusions

We believe that an initial assessment of the patient considering the severity of illness may allow a better allocation of resources through targeted individualized standard treatments, which can be adapted to the characteristics of the specific patient. Such an approach would lead, for example, to avoidance of establishing therapeutic/rehabilitative strategies that the patient is not able to deal with; on the contrary, it could instead foster the search for specific treatments for patients with negative symptoms, less responsive to the most common treatments.

The recovery style does not turn out to be an indicator of the financial commitment expected for a specific patient. However, we suggest that it should be included in a broader assessment of the subject, together with the expression and severity of the illness and social functioning. If used in this context, the recovery style should be able to provide some general guidelines: in presence of the same severity of illness, an integrative recovery style may portend a greater engagement of services and so a higher spending, compared to the use of sealing-over.

## Figures and Tables

**Table 1:** Frequency of recovery style patterns according to ISOR/RSQ results and *t*-test for annual cost per patient between different groups of recovery style.

Recovery style	%	Mean	Standard deviation	*t*-test *P*-value
ISOS				
Integration	60.4	6321.54	±6962.38	0.580
Sealing over	39.6	5338.15	±4657.35	
RSQ				
Integration	79.2	6001.46	±6399.36	0.897
Sealing over	20.8	5726.59	±5366.76	

**Table 2:** Pearson correlation between annual cost and recovery style/PANSS subscale. We did not find any significant correlation with recovery style scales. We instead highlighted a positive correlation between annual cost and PANSS-G and PANSS-P subscales.

Variable	ISOS	RSQ	PANSS-G	PANSS-N	PANSS-P
Annual cost					
Pearson correlation	−0.079	−0.018	0.452	0.203	0.333
*P*-value	0.580	0.897	**0.001**	0.150	**0.016**

Bold font refers to statistical significance (*P*-value less than 0.05).

**Table 3:** Means scores of ISOS, RSQ, and PANSS subscales in the four engagement groups are reported. In the last column ANOVA significance values are listed.

Variable	Mean				ANOVA *P*-value
	Group I	Group II	Group III	Group IV	
ISOS	3.6	3.23	3	3.13	0.761
RSQ	7.8	8.55	7.40	8.40	0.666
PANSS-G	35.2	32.68	44.80	48.13	**0.003**
PANSS-P	13.6	15.32	22.80	18.40	0.074
PANSS-N	22	16.68	24.60	22.80	**0.049**
Annual cost	1352.22	4707.67	8800.37	10120.16	**0.001**

Bold font refers to statistical significance (*P*-value less than 0.05).

**Table 4:** Pearson Bivariate correlation between service engagement, PANSS subscales and recovery style.

Variable	ISOS	RSQ	PANSS-G	PANSS-N	PANSS-P	Annual cost
Service engagement						
Pearson correlation	−0.146	−0.012	0.447	0.175	0.276	0.530
*P*-value	0.302	0.931	**0.001**	0.214	**0.047**	**0.000**

Bold font refers to statistical significance (*P*-value less than 0.05).

## References

[1] Fenton WS (2003). Shared decision making: a model for the physician-patient relationship in the 21st century?. *Acta Psychiatrica Scandinavica*.

[2] Hamann J, Leucht S, Kissling W (2003). Shared decision making in psychiatry. *Acta Psychiatrica Scandinavica*.

[3] Noble L (1998). Doctor-patient communication and adherence to treatment. *Adherence to Treatment in Medical Conditions*.

[4] World Health Organization (2003). *Adherence to Long Term Therapies: Evidence for Action*.

[5] Gilbert JR, Evans CE, Haynes RB, Tugwell P (1980). Predicting compliance with a regimen of digoxin therapy in family practice. *Canadian Medical Association Journal*.

[6] Stephenson BJ, Rowe BH, Haynes RB, Macharia WM, Leon G (1993). Is this patient taking the treatment as prescribed?. *Journal of the American Medical Association*.

[7] Scott J (1999). Cognitive and behavioural approaches to medication adherence. *Advances in Psychiatric Treatment*.

[8] Tacchi MJ, Scott J (2005). *Improving Adherence in Schizophrenia and Bipolar Disorder*.

[9] Horvath AO, Greenberg LS (1989). Development and validation of the working alliance inventory. *Journal of Counseling Psychology*.

[10] Frank AF, Gunderson JG (1990). The role of the therapeutic alliance in the treatment of schizophrenia. Relationship to course and outcome. *Archives of General Psychiatry*.

[11] Tait L, Birchwood M, Trower P (2002). A new scale (SES) to measure engagement with community mental health services. *Journal of Mental Health*.

[12] O’Brien A, White S, Fahmy R, Singh SP (2009). The development and validation of the SOLES, a new scale measuring engagement with mental health services in people with psychosis. *Journal of Mental Health*.

[13] Startup M, Wilding N, Startup S (2006). Patient treatment adherence in cognitive behaviour therapy for acute psychosis: the role of recovery style and working alliance. *Behavioural and Cognitive Psychotherapy*.

[14] Poloni N, Callegari C, Buzzi A (2010). The Italian version of ISOS and RSQ, two suitable scales for investigating recovery style from psychosis. *Epidemiologia e Psichiatria Sociale*.

[15] McGlashan TH, Levy ST, Carpenter WT (1975). Integration and sealing over. Clinically distinct recovery styles from schizophrenia. *Archives of General Psychiatry*.

[16] McGlashan TH, Levy ST (1977). Sealing over in a therapeutic community. *Psychiatry*.

[17] McGlashan TH (1987). Recovery style from mental illness and long-term outcome. *Journal of Nervous and Mental Disease*.

[18] Drayton M, Birchwood M, Trower P (1998). Early attachment experience and recovery from psychosis. *British Journal of Clinical Psychology*.

[19] Thompson KN, McGorry PD, Harrigan SM (2003). Recovery style and outcome in first-episode psychosis. *Schizophrenia Research*.

[20] Tait L, Birchwood M, Trower P (2003). Predicting engagement with services for psychosis: insight, symptoms and recovery style. *British Journal of Psychiatry*.

[21] Tait L, Birchwood M, Trower P (2004). Adapting to the challenge of psychosis: personal resilience and the use of sealing-over (avoidant) coping strategies. *British Journal of Psychiatry*.

[22] Modestin J, Soult J, Malti T (2004). Correlates of coping styles in psychotic illness. *Psychopathology*.

[23] World Health Organization (1992). *Manual of the International Classification of Diseases, Injuries, and Causes of Death*.

[24] Percudani M, Belloni G, Contini A, Barbui C (2002). Monitoring community psychiatric services in Italy: differences between patients who leave care and those who stay in treatment. *British Journal of Psychiatry*.

[25] Kay SR, Fiszbein A, Opler LA (1987). The positive and negative syndrome scale (PANSS) for schizophrenia. *Schizophrenia Bulletin*.

[26] Pancheri P, Brugnoli R, Carilli L, Delle Chiaie R, Marconi PL, Petrucci RM (1995). Valutazione dimensionale della sintomatologia schizofrenica. Validazione della versione italiana della Scala per la valutazione dei Sintomi Positivi e Negativi (PANSS). *Giornale Italiana Psicopatologia*.

[27] Purnine DM, Carey KB, Maisto SA, Carey MP (2000). Assessing positive and negative symptoms in outpatients with schizophrenia and mood disorders. *Journal of Nervous and Mental Disease*.

[28] Donisi V, Jones J, Pertile R (2011). The difficult task of predicting the costs of community-based mental health care. A comprehensive case register study. *Epidemiology and Psychiatric Sciences*.

[29] Galderisi S, Maj M (2009). Deficit schizophrenia: an overview of clinical, biological and treatment aspects. *European Psychiatry*.

[30] Kirkpatrick B, Galderisi S (2008). Deficit schizophrenia: an update. *World Psychiatry*.

